# Reproducibility of Three-Dimensional Facial Surface Models Generated from Repeated CT and CBCT Scans: An Ex Vivo Study

**DOI:** 10.3390/diagnostics16142136

**Published:** 2026-07-08

**Authors:** Mohammed Ghamri, Mimmi Sunnard, Konstantinos Dritsas, Ragda Alamoudi, Simos Psomiadis, Demetrios Halazonetis, Nikolaos Gkantidis

**Affiliations:** 1 Department of Orthodontics and Dentofacial Orthopedics, School of Dental Medicine, University of Bern, CH-3010 Bern, Switzerland; mk.ghamri@hotmail.com (M.G.); mimmi.sunnard@unibe.ch (M.S.); konstantinos.dritsas@unibe.ch (K.D.); 2 North Jeddah Speciality Dental Center, King Abdullah Medical Complex, Jeddah Second Health Cluster, Ministry of Health, Jeddah 23816, Saudi Arabia; 3 Dentistry Program, Division of Orthodontics, Batterjee Medical College, Jeddah 21442, Saudi Arabia; raghad.alamoudi93@outlook.com; 4 Department of Oral and Maxillofacial Surgery, School of Dentistry, National and Kapodistrian University of Athens, 11527 Athens, Greece; simospsomiadis@gmail.com; 5 Department of Orthodontics, School of Dentistry, National and Kapodistrian University of Athens, 11527 Athens, Greece; dhalaz@dent.uoa.gr

**Keywords:** reproducibility of results, face, imaging, three-dimensional, cone-beam computed tomography, tomography, X-ray computed, models, anatomic

## Abstract

**Background/Objectives**: This study aimed to evaluate the reproducibility of 3D facial skeletal surface models generated from repeated computed tomography (CT) and cone bean CT (CBCT) scans. **Methods**: Four hydrated-dry skull specimens, with soft-tissue simulation using water, were scanned twice within seconds using a single CT scanner. Eight skulls were scanned twice using two CBCT scanners with different settings. An experienced operator segmented all facial skeletal surfaces using a visually estimated optimal threshold. 3D models from repeated scans were superimposed on the forehead, zygomatic area, and maxilla using a best-fit algorithm. Deviations between superimposed models were assessed through distances in predefined areas and color-coded maps, attributed to segmentation errors or tomographic volume generation inaccuracies. Two facial surfaces from each acquisition setting with the largest deviations were resegmented using the original threshold value. **Results**: Repeated threshold determinations showed no significant differences (*p* = 0.266; median difference: −6.0, IQR: 39.5). Significant differences were noted between CT and CBCT scanners, but not among CBCT scanners. The median Mean Absolute Distance (MAD) for CBCT was 0.059 mm (IQR: 0.032) versus 0.016 mm (IQR: 0.007) for CT. Color-coded maps confirmed higher consistency in CT and Newtom models, with the low-radiation Planmeca protocol achieving comparable reproducibility. Differences primarily arose from image generation parameters, not threshold estimation. **Conclusions**: CT provides slightly more consistent 3D facial skeletal surface models. However, CBCT scanners, including those using low-radiation protocols, also demonstrate high reproducibility, reinforcing their reliability in diagnosing and planning treatment for facial morphology variations.

## 1. Introduction

The use of three-dimensional (3D) imaging in dentistry is increasing [1,2], as it reflects the inherent 3D nature of the depicted structures. Advances in technology have significantly reduced the additional radiation exposure associated with tomographic 3D imaging [3], improving the cost–benefit ratio for patients and making it a more favorable option for diagnostics and treatment planning. Computed tomography (CT) reduces the risks for operator-related errors during image analysis, as there is no overlap of anatomical structures and the orientation-related errors are minimized [4].

The reliability of the acquisitions and the quality of the images determine their diagnostic value. Ideally, they must correspond to the original morphology of the patient. In dentistry, CTs are typically generated using cone beam CT (CBCT) scanners, as they offer reduced radiation exposure at a reasonable size and cost. Imaging systems perform differently, with acquisition parameters like voxel size, kV, mA, and field of view (FOV) significantly affecting image quality and radiation dose [5]. A recent study [6] assessed the trueness of computed tomography derived facial skeletal surface models from one CT scanner (Revolution CT) and two CBCT scanners (Newtom and Planmeca), also including a low-dose setting. The tomographically derived models were superimposed and compared with a gold standard model created by a high-accuracy optical 3D surface scanner (Artec Space Spider, Artec3D, Luxembourg). The results were promising, with deviations of 0.12 mm on average, and no significant differences between the tomographic scanners or settings [6].

Traditional CT scans are known for their high consistency due to their precise control of scanning parameters and the standardized nature of the technology. They utilize a fan-shaped X-ray beam that rotates around the patient, capturing images slice by slice [7]. CBCT uses a cone-shaped X-ray beam to capture volumetric data in a single rotation. The consistency of CBCT images can be influenced by various factors, including patient movement, positioning, scanner settings, and the cone beam capturing different parts of the head at each rotation position [8,9,10]. Additionally, CBCT images are prone to artefacts due to higher noise levels, scatter, and geometric errors [11]. Another limitation of CBCT is the use of grayscale values rather than the standardized Hounsfield units provided by CT scans [12,13]. Compared with CT, CBCT image quality may be more susceptible to acquisition and reconstruction-related variability, potentially affecting reproducibility. While encouraging trueness outcomes (comparison with a reference standard) have been reported previously using the same experimental material [6,14], precision represents a distinct measurement property that had not yet been evaluated. Both properties are required for a comprehensive assessment of imaging performance in diagnosing craniofacial morphology. Precision refers to the degree of consistency and reproducibility of repeatedly acquired surface models under the same conditions (ISO 5725-1) and represents a property complementary to trueness. Therefore, the present study aimed to evaluate the reproducibility of repeated CT and CBCT scans and to determine the extent to which scanner-related variability may influence facial skeletal surface model generation.

## 2. Materials and Methods

### 2.1. Ethical Approval

The ethics committee of the Dental School of the National and Kapodistrian University of Athens approved the project protocol (Protocol number: 335, Date of approval: 2 May 2017, Renewed on 16 November 2021).

### 2.2. Material

The skull sample, acquisition protocols, and segmentation procedures have been described previously in studies evaluating the trueness of CT- and CBCT-derived craniofacial surface models [6,14]. The present investigation addresses a distinct research question. Whereas previous studies compared tomographic models with an external reference standard to assess accuracy, the current study evaluates reproducibility by comparing independently acquired repeated scans of the same specimens. Key methodological aspects are repeated here to facilitate understanding of the study. Eight dry skulls from the Municipal Cemetery of Serres, Greece, were used. Approval was obtained from the local authorities (Municipality of Serres, Greece, Protocol Number: 4044/12.07.2018). At the time of acquisition, the identities of the specimens were unknown. Therefore, informed consent was not applicable. The sample size was determined based on empirical data [15], as well as the availability of resources and the authors’ research experience. Because of logistical constraints and limited access to medical CT facilities, four skulls were scanned with CT, whereas all eight skulls were scanned with the CBCT systems. Mini-screw-anchored expansion appliances were present in the palates of all skulls as part of a separate project. Consequently, metal artifacts were present in the tomographic images, primarily affecting the maxillary alveolar region.

### 2.3. Image Acquisition

The whole skulls were scanned twice using four different acquisition settings. The second scanning was performed immediately after the first, with all parameters held constant. The specimens were not intentionally repositioned between repeated scans. However, minor positional changes may have occurred due to scanner vibrations, occasional readjustment by the radiologist when deemed necessary, and the water-embedding setup, in which the skulls were supported but not rigidly fixed. Tomographic images were acquired under hydrated conditions to simulate soft tissues [16,17,18]. Soft-tissue simulation was achieved by enclosing each specimen in an artificial 3D-printed head shell (PETG, MasterFill Premium PETG Pro, 3DHUB, Athens, Greece) filled with water, as described in previous studies [6,19]. The skull was centered within the head shell using radiolucent water-absorbing sponges to ensure realistic soft-tissue thickness.

The specimens underwent repeated full head tomography scans with the following CT and CBCT scanners and acquisition settings [6]:

1. CT scanner (Revolution CT 256, GE Healthcare, Chicago, IL, USA; 251 Hellenic Airforce Hospital, Athens, Greece). kV: 120, mA: 490 in the area of interest (automatically configured based on tissue mass and density), exposure time: 1 s, slice thickness: 0.625 mm, voxel size: 0.49 to 0.62 × 0.49 to 0.62 × 0.31 (interslice) mm, FOV: full head (displayed FOV: 25 cm).
2. CBCT scanner I (Newtom VGiMK4, Verona, Italy; Dental School, National and Kapodistrian University of Athens Greece). kV: 110, mA: 4–5 (automatically configured based on tissue mass and density), exposure time: 4 s, voxel size: 0.3 × 0.3 × 0.3 mm, FOV: ⌀15 × 15 cm.
3. CBCT scanner II—regular dose settings (Planmeca F, Planmeca Promax 3D Mid 2018 (Planmeca, Helsinki, Finland); Digital Iatriki Apeikonisi, Athens, Greece). kV: 100, mA: 8, exposure time: 12 s, voxel size: 0.2 × 0.2 × 0.2 mm, FOV: ⌀20 × 17 cm.
4. CBCT scanner II—ultra-low dose settings (Planmeca U). kV: 100, mA: 8, exposure time: 6 s, voxel size: 0.2 × 0.2 × 0.2 mm, FOV: ⌀20 × 17 cm.

All scans were performed by professional radiologists under standardized conditions. Sample tomographic slices with each acquisition setting have been published previously [6].

### 2.4. Tomographic Data Segmentation

All tomographic images were exported in DICOM format and imported into Viewbox 4 (dHAL Software, Kifissia, Greece). The data were processed by a single experienced operator (M.G.), who performed bone segmentation of the facial structures by visually selecting an optimal threshold. The threshold was iteratively adjusted until the segmented contour corresponded visually to the apparent cortical bone boundary. To optimize this process, the contour line corresponding to each threshold was manually aligned with the outer limit of the skeletal surface of interest across multiple 2D tomographic slices. The final threshold was determined after several adjustments, aiming to achieve optimal segmentation of the entire facial model based on the operator’s visual assessment. Each defined threshold was documented in a Microsoft Excel sheet (Microsoft Corporation, Redmond, WA, USA). The same process of single threshold selection was repeated independently after a two-week interval for the volumes obtained from the second tomographic scans. Using the defined thresholds, a variant of the marching cubes algorithm [20] was applied to create dense triangular mesh models, which were afterwards saved as STL files. These models consisted of approximately 900,000 vertices for CT scanner, 1,600,000 for Newtom scanner, and 4,000,000 for Planmeca scanner-generated volumes [6].

### 2.5. Reproducibility of the Directly Repeated Craniofacial Scans

**Figure 1:**
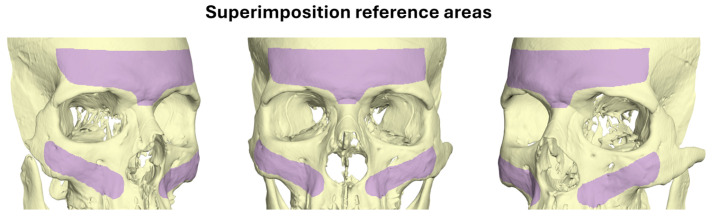
**Superimposition reference areas (marked in purple).** A variant of the iterative closest point (ICP) algorithm was applied on the marked surfaces for the best-fit approximation of repeatedly generated facial surface models.

Each pair of the visually segmented surface models derived from the repeated scans was superimposed using a variant of the iterative closest point (ICP) algorithm [21], with the following software settings: 100% estimated overlap of meshes (85–90% overlap in one CT-, one Newtom-, one Planmeca U-, and two Planmeca F-scan derived surfaces that had few missing structures at the superimposition reference areas), point-to-plane matching, exact nearest neighbor search, 100% point sampling, and 50 iterations. Figure 1 illustrates the superimposition reference area used for this.

**Figure 2:**
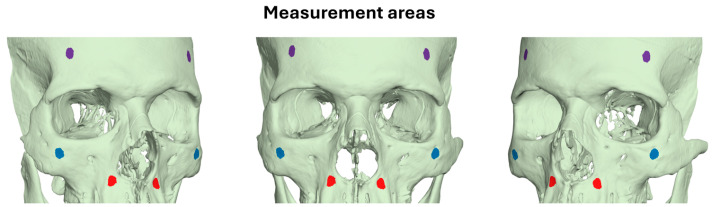
**Three measurement areas depicted as differently colored circular regions.** Each measurement area consisted of two bilateral markers of the same color, with each marker covering a surface area of 30 mm^2^.

For reproducibility testing, the congruence of the best-fit approximated repeatedly acquired model pairs was evaluated [6,9] using the mean absolute distance (MAD) and the standard deviation of the absolute distances (SDAD) between the models. This involved measuring the distance from each vertex point of one mesh model to the closest point on the second mesh, in three predefined measurement areas, each having an extent of 30 mm^2^ and comprising approximately 2000 triangles. The measurement areas were located bilaterally on the forehead, the zygomatic process, and the maxillary complex. Each anatomical structure’s bilateral selection was considered as a single measurement area (Figure 2). Color-coded distance maps over the entire surface models were also generated. Zero distance between the superimposed models indicated perfect reproducibility.

### 2.6. Reproducibility of the Repeatedly Acquired Surface Models, Segmented Through the Same Threshold

To investigate the impact of threshold estimation on reproducibility outcomes, we selected the two pairs of scans from each acquisition setting that exhibited the lowest reproducibility following independent single-threshold segmentations. These scans were re-segmented using the same threshold value applied during the initial segmentation. The resulting models were then best-fit approximated as described earlier, and color-coded distance maps were generated.

### 2.7. Statistical Analysis

Data were tested for normality through Shapiro–Wilk test and were not always normally distributed. Based on these outcomes, primarily non-parametric statistics were applied in the study.

Systematic differences between visually defined segmentation thresholds on repeated tomographic scans were tested using one-sample *t*-test. Differences between tomographic imaging systems regarding the same outcomes were tested through Kruskal–Wallis test.

Differences in the MAD and SDAD between superimposed repeatedly acquired models, through the four settings were tested using Kruskal–Wallis test, followed by Mann–Whitney U test for pairwise comparisons (significance levels adjusted by the Bonferroni correction), if applicable. In these tests, all measurement areas per acquisition setting were either considered one variable or each measurement area was tested individually as a single variable.

## 3. Results

### 3.1. Intra-Operator Reproducibility of the Visually Defined Segmentation Threshold

**Figure 3:**
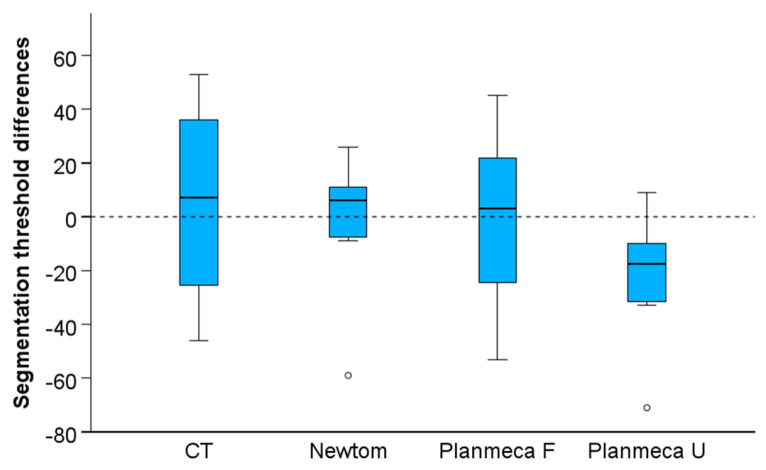
**Box plots showing the intra-operator reproducibility of the visually determined segmentation thresholds.** Outliers are shown as black circles (further from the median more than 1.5 times the IQR). A difference of 10 in threshold values represents 0.25% of the full range of voxel values in the CT images, 0.22% in the Newtom images, and 0.29% in the Planmeca images.

The differences between the repeatedly selected segmentation threshold values were not significantly different from zero (*n* = 28, *p* = 0.266; Small effect size: Cohen’s d = −0.214, 95%CI: −0.587, 0.162). The median difference between repeated threshold values was small (median: −6.0, IQR: 39.5) and did not differ between imaging systems (Kruskal–Wallis test: *p* = 0.162, Figure 3).

### 3.2. Reproducibility of the Repeatedly Acquired and Segmented Surface Models

**Figure 4:**
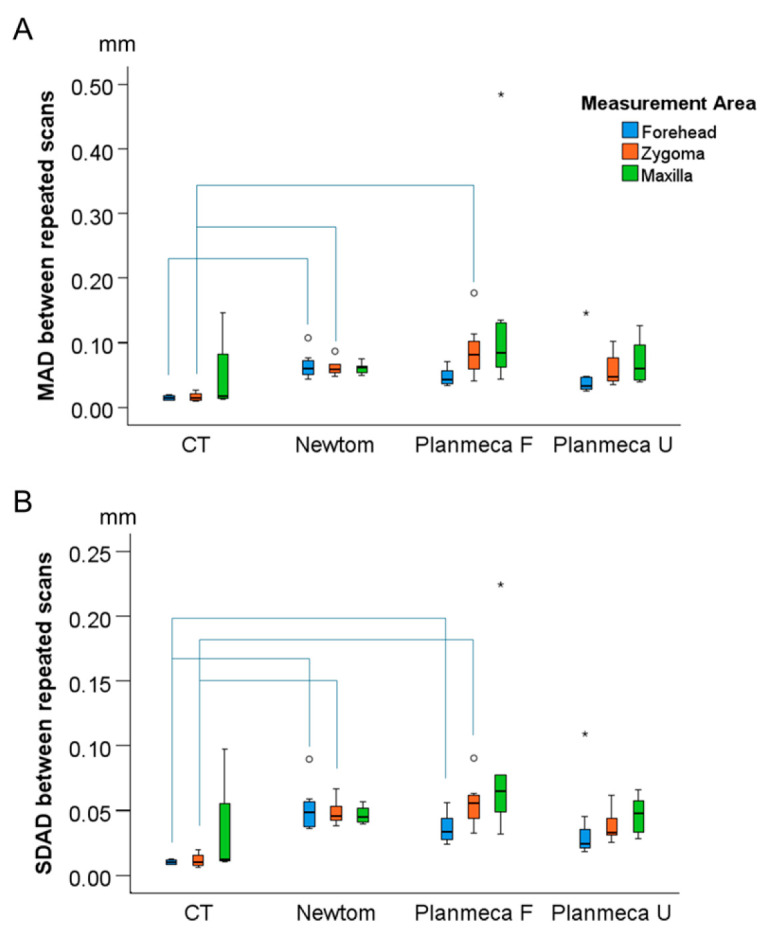
**Differences between repeatedly acquired and segmented surface models at three measurement areas.** Box plot showing (**A**) the Mean Absolute Distance (MAD) values and (**B**) the Standard Deviations of the absolute distances (SDAD) values. The lines connect variables that show significant differences (*p* < 0.05) detected through Kruskal–Wallis, followed by Mann–Whitney U test (Bonferroni adjusted). Outliers are shown as black circles (further from the median more than 1.5 times the IQR) or asterisks in more extreme cases (further from the median more than 3 times the IQR).

The overall reproducibility of the craniofacial scans was high, with differences between superimposed surface models consistently being lower than 0.2 mm (Figure 4). There was only an extreme outlier for the Planmeca Full acquisition (maxilla MAD = 0.484 mm, SDAD = 0.053 mm). When all measurement areas were considered as one variable, there were significant differences between tomographic imaging systems (Kruskal–Wallis test: *p* < 0.001). Pairwise comparisons revealed significant differences between CT and all CBCT scans, but not among CBCT scans (Table 1). The overall median MAD for CBCT scans was 0.059 mm (IQR: 0.032) (Newtom, median: 0.060, IQR: 0.015; Planmeca F, median: 0.068, IQR: 0.051; Planmeca U, median: 0.045, IQR: 0.043), compared to 0.016 mm (IQR: 0.007) for CT. When each measurement area was analyzed as a separate variable, there were very few significant differences identified between the CT and CBCT scanning systems (MAD; CT vs. Newtom Forehead: *p* = 0.001, Zygoma: *p* = 0.044; CT vs. Planmeca F Zygoma: *p* = 0.003) (Figure 4A).

The overall SDAD between repeated craniofacial scans was small, with almost all values being lower than 0.1 mm (Figure 4B). There was only an extreme outlier value for the Planmeca Full acquisition (maxilla MAD = 0.127 mm, SDAD = 0.224 mm). When all measurement areas were considered as one variable, there were significant differences between tomographic imaging systems (Kruskal–Wallis test: *p* < 0.001). Pairwise comparisons demonstrated significant differences between CT and CBCT scans, while no differences were observed among the CBCT scans (Table 1). The overall median SDAD for all CBCTs was 0.046 mm (IQR: 0.023) (Newtom, median: 0.046, IQR: 0.014; Planmeca F, median: 0.054, IQR: 0.030; Planmeca U, median: 0.033, IQR: 0.023), compared to 0.011 mm (IQR: 0.004) for the CT. When each measurement area was analyzed as a separate variable, there were few significant differences identified between the CT and CBCT scans (SDAD; CT vs. Newtom Forehead: *p* = 0.001, Zygoma: *p* = 0.017; CT vs. Planmeca F Forehead: *p* = 0.041, Zygoma: *p* = 0.003) (Figure 4B).

**Figure 5:**
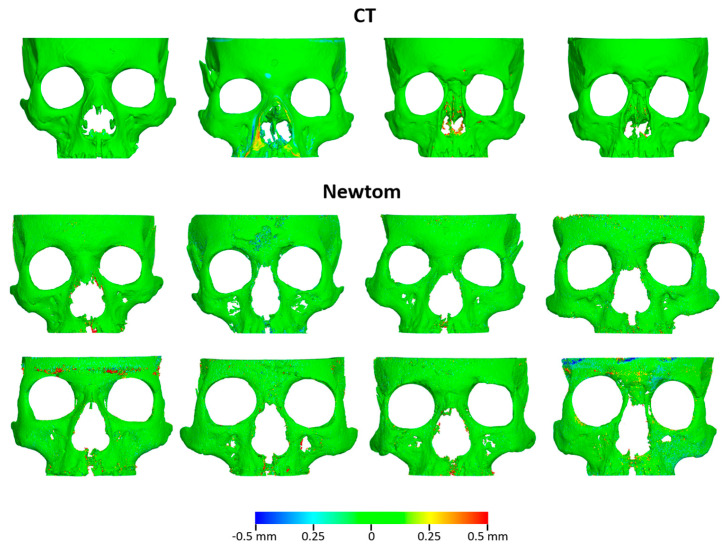
**Differences between repeatedly acquired tomographic scans with CT and Newtom scanners.** Color-coded distance maps between best-fit approximated corresponding facial surface models, segmented from repeated scans. Segmentations were performed using operator-defined thresholds, independently determined for each segmentation.

**Figure 6:**
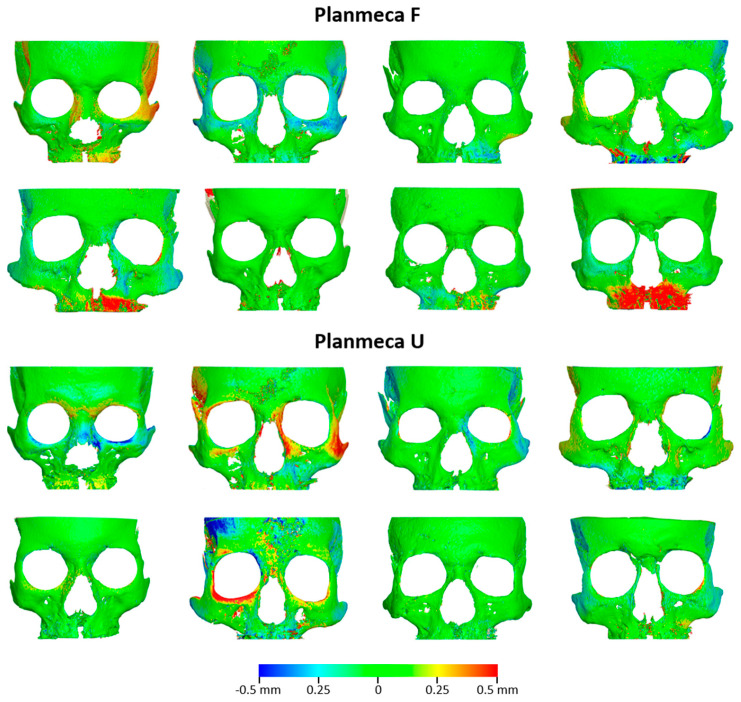
**Differences between repeatedly acquired tomographic scans with Planmeca F and Planmeca U settings.** Color-coded distance maps between best-fit approximated corresponding facial surface models, segmented from repeated scans. Segmentations were performed using operator-defined thresholds, independently determined for each segmentation.

In agreement with the measurement data depicted in Figure 4, the color-coded distance maps between the superimposed surface models revealed consistently high reproducibility for CT and Newtom scanners (Figure 5). Slightly higher deviations were detected for the Planmeca unit, with two out of eight cases in each setting illustrating differences up to 0.5 mm at few facial areas (Figure 6; Planmeca F: Skulls 6 and 16, Planmeca U: Skulls 4 and 9A). Deviations in areas other than the outer facial surfaces are not relevant, as they were not included in the segmentation threshold definition or used as superimposition references for the best-fit model approximation.

### 3.3. Reproducibility of the Repeatedly Acquired Surface Models, Segmented Through the Same Threshold

**Figure 7:**
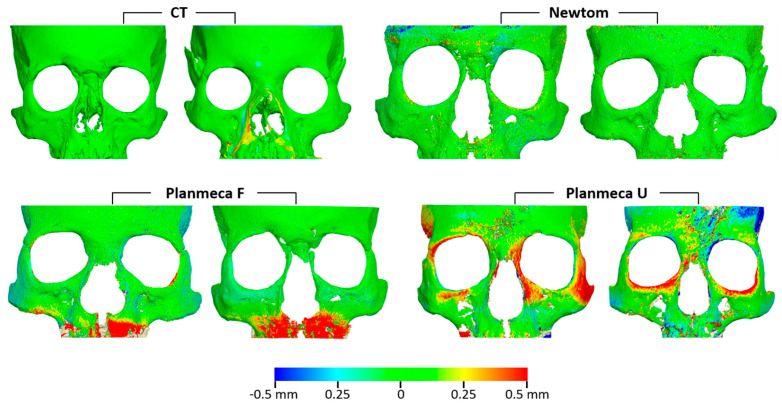
**Differences between facial surface models segmented from repeatedly acquired tomographic scans using the same threshold.** The images depict color-coded distance maps between best-fit approximated models, segmented from the two scans with the lowest reproducibility for each acquisition setting, based on independent single-threshold segmentations.

The color-coded distance maps between repeatedly acquired surface models segmented with the same threshold, showed similar differences to those of surface models generated using different thresholds (Figure 7). This indicates that the error due to threshold selection is small.

## 4. Discussion

In the present study, we aimed to assess the precision of CT and CBCT imaging systems in terms of reproducibility in facial surface model generation from directly repeated scans. We compared various scanners and evaluated the Planmeca CBCT scanner at two dose settings to assess if low-dose exposure maintains comparable precision. Four skulls were repeatedly scanned with CT, while eight were used for CBCT. The smaller CT sample was a consequence of the limited availability of medical CT imaging resources for research purposes. Nevertheless, CT imaging is generally considered to provide highly consistent image acquisition and reconstruction [22,23], which may have mitigated the impact of the smaller sample size. Tomographic images were acquired with water embedding to simulate soft tissues [18,19], as studies have shown that imaging without soft tissues has significant limitations [8,24,25,26,27,28,29,30]. The overall reproducibility of repeated craniofacial scans was high, with nearly all differences measuring less than 0.2 mm. The only notable outlier occurred in the maxillary measurements of the Planmeca Full acquisition and may have been related to metal artifacts generated by the mini-screw–anchored expansion appliances.

Although threshold selection is crucial for tomographic volume segmentation [9], studies show that visually defined single-threshold segmentation does not compromise the accuracy of skeletal facial surface models [6,31]. Thicker bone is less sensitive to threshold variations, while thinner structures, such as the maxilla, are more affected. This study evaluated visual segmentation effects. Differences in repeatedly defined thresholds by the same operator were negligible (<0.3% of each system’s voxel range), consistent with prior reports showing differences smaller than 50 μm [6]. Re-segmenting the least reproducible scans with identical thresholds produced similar variations to those observed with different thresholds. The experienced operator’s role suggests minimal segmentation impact, with differences likely due to imaging system parameters. Potential sources of error include hardware malfunctions (e.g., X-ray tube issues, detector errors, or mechanical instability), which can introduce image blurring or misalignment, and software-related factors. Outdated software or poorly calibrated image processing systems may lead to poor contrast, incorrect grayscale calibration, or improper image sharpening [32,33,34]. Reconstruction algorithms, such as the widely used FDK (Feldkamp, Davis, and Kress), which relies on filtered back-projection (an analytical algorithm), or iterative algorithms, may contribute to variation [35], though this should not affect within-scanner comparisons.

Our primary aim was to assess the reproducibility of surface models with different tomographic acquisition systems. When all measurement areas were considered together, significant differences were found between CT and CBCT but not among CBCT scanners or settings. The overall median MAD was 0.059 mm for CBCT and 0.016 mm for CT. These values represent the combined effects of the tomographic acquisition and reconstruction process together with segmentation variability. To estimate the relative contribution of the tomographic imaging process, we compared these values with segmentation reproducibility previously quantified on the same specimens under identical experimental conditions [6]. In that study, segmentation error was limited to 0.004 mm (IQR: 0.005) for CBCT and 0.006 mm (IQR: 0.007) for CT. Under the assumption that segmentation variability and tomographic acquisition variability are independent, the remaining reproducibility error can be interpreted as an approximate estimate of scanner-related variability, corresponding to 0.053 mm for CBCT and 0.010 mm for CT. Although this represents an indirect estimate of scanner error, it indicates that the contribution of the tomographic imaging process is greater than that of visual segmentation, particularly for CBCT. These findings further suggest that, when performed by an experienced operator, visual threshold-based segmentation is not a major source of variability. Segmentation error remained below 0.02 mm, even when repeatedly segmented models retained their original spatial relationship within the source radiographic volume [6]. Although CT demonstrated significantly higher reproducibility than CBCT, the magnitude of the difference was very small (median difference approximately 0.04 mm) and substantially smaller than the magnitude generally considered clinically relevant for craniofacial morphology diagnosis, treatment planning, or longitudinal assessment. Therefore, despite statistically significant differences, all evaluated CBCT protocols demonstrated reproducibility that is unlikely to meaningfully affect clinical or research applications. The superior precision of CT was expected due to fundamental differences between modalities. CT captures thin slices sequentially with standardized processing, using advanced algorithms for accurate diagnostics. In contrast, CBCT uses a cone-shaped X-ray beam to capture the entire volume in one rotation, relying on simpler reconstruction algorithms such as Feldkamp–Davis–Kress (FDK) [6,7,9]. While computationally efficient, FDK is sensitive to fewer projections, more prone to image distortions, and less effective at reducing noise, potentially affecting reconstruction quality [35,36]. Additionally, CBCT captures different head regions at each rotation, unlike CT, which maintains consistent full-volume coverage.

This study evaluated different tomographic imaging systems, including a standard-dose and a low-dose Planmeca CBCT protocol, both defined by specialist radiologists for craniofacial morphology assessment. The low-dose protocol demonstrated precision comparable to the regular setting, reducing radiation risks, which is crucial for sensitive populations like children, where CBCT use in orthodontics is increasing [37,38,39]. Despite its advantages, a high-field-of-view, low-dose CBCT still requires more radiation than a combination of panoramic and cephalometric radiographs, making it unsuitable for routine orthodontic diagnosis [40]. However, if lower-dose CBCT achieves precision and trueness [6] comparable to current standards while maintaining radiation levels similar to or below conventional 2D imaging, it could replace multiple 2D radiographs with a single, more informative 3D scan [41]. Further dose reduction is possible by limiting the field of view and optimizing scan parameters [15,40,42,43], enhancing both safety and diagnostic accuracy. These findings specifically apply to craniofacial morphology diagnostics and may not extend to other applications such as caries detection, fine bone assessments, or root morphology analysis.

A previous study assessed segmentation errors using serial CBCT images of actual patients [31]. Researchers compared errors between two manual threshold inputs and one manual versus one automatic input, where the software standardized the threshold. Both methods showed satisfactory reproducibility with minimal error. The median segmentation error for manually repeated thresholds ranged from 0.07 to 0.12 mm, consistent with prior findings in the current sample [6], confirming segmentation reliability across sessions. That study [6] found a maximum difference of 0.2 mm between repeatedly segmented facial surface models, while the present study, which also accounted for hardware and software errors, showed a maximum of 0.5 mm. This suggests a tomographic system error of 0.3 mm. Few studies evaluate segmentation errors using manual thresholds, as most rely on automatic thresholding or omit segmentation error analysis. However, assessing these errors is crucial for defining clinical applications and ensuring accurate modelling [44,45].

This study follows a well-established methodology, including rigorous testing of hardware and software for 3D imaging, superimposition, and assessment, ensuring reliable outcomes [6,9,14,15,31,46]. A key strength is the comparison of multiple tomographic scanners using acquisition protocols tailored by radiology specialists to reflect real-world clinical practices. Our findings align with previous research [6], demonstrating that the tested low-radiation protocol achieves trueness comparable to standard protocols. Additionally, it offers reduced radiation exposure without compromising precision.

A key limitation of this study is the use of ex vivo skulls instead of patient data. While water embedding simulates soft tissues and the skulls were no rigidly fixed, real scans may differ due to motion artifacts [47] and other scanner-related errors affecting image quality and reproducibility. Thus, these findings may not fully reflect clinical complexities, especially in challenging cases. However, using ex vivo skulls allowed for a safe, ethical assessment of tomographic scan reproducibility without patient risk. Furthermore, all skulls contained mini-screw-anchored expansion appliances, which generated metal artifacts in the maxillary region. Although these artifacts may have contributed to the isolated outlier observed in the maxillary measurements, they were present in all acquisitions and therefore reflected a realistic imaging scenario. Despite their presence, reproducibility of the facial surface area remained high across all imaging systems. Another limitation is the unequal sample size between CT and CBCT acquisitions. Despite the high image consistency of CT scans, because only four skulls were scanned with CT, comparisons between imaging modalities should be interpreted cautiously and confirmed in larger studies. Finally, although the present study used a single experienced operator to minimize segmentation-related variability, recent deep learning-based segmentation methods have demonstrated high accuracy across CT and CBCT acquisition protocols, substantially reducing operator dependency and facilitating standardized workflows across different users [48].

## 5. Conclusions

The reproducibility of repeated craniofacial scans was high, with CT demonstrating significantly higher reproducibility (median error: 0.02 mm) than all CBCT protocols. Nevertheless, the magnitude of these differences was very small, and all CBCT systems exhibited high reproducibility (median error: 0.06 mm). These findings provide additional support to the use of low-dose CBCT imaging for facial skeletal morphology assessment, as high reproducibility was maintained despite reduced radiation exposure. Repeated segmentations using the same threshold indicated that differences stemmed from image generation parameters rather than segmentation.

## Figures and Tables

**Table 1:** Pairwise comparisons between tomographic machines regarding the MADs between best-fit approximated repeated craniofacial scans.

MAD	Test Statistic	Std. Error	Std. Test Statistic	Sig.	Adj. Sig. ^a^
CT vs. Planmeca U	−27.42	8.62	−3.18	0.001	0.009
CT vs. Newtom	−37.87	8.62	−4.39	<0.001	0.000
CT vs. Planmeca F	−40.00	8.62	−4.64	<0.001	0.000
Planmeca U vs. Newtom	10.46	7.04	1.48	0.137	0.825
Planmeca U vs. Planmeca F	12.58	7.04	1.79	0.074	0.444
Newtom vs. Planmeca F	−2.12	7.04	−0.30	0.763	1.000
SDAD					
CT vs. Planmeca U	−25.25	8.62	−2.93	0.003	0.020
CT vs. Newtom	−39.52	8.62	−4.58	<0.001	0.000
CT vs. Planmeca F	−40.52	8.62	−4.70	<0.001	0.000
Planmeca U vs. Newtom	14.27	7.04	2.03	0.043	0.256
Planmeca U vs. Planmeca F	15.27	7.04	2.17	0.030	0.181
Newtom vs. Planmeca F	−1.00	7.04	−0.14	0.887	1.000

Asymptotic significances (2-sided tests) are displayed. The significance level is 0.05. ^a^ Significance values have been adjusted by the Bonferroni correction for multiple tests.

## Data Availability

The raw data supporting the conclusions of this article will be made available by the authors on request.
